# Betting on Australian Rules Football: Can Expert Tipsters beat Randomness?

**DOI:** 10.1007/s10899-023-10244-9

**Published:** 2023-08-07

**Authors:** Ben J. Riley, Lee Li, David Plevin, Michael Baigent

**Affiliations:** 1 College of Medicine and Public Health, Flinders University, South, Australia; 2 Adelaide Medical School Discipline of Psychiatry Adelaide University, South, Australia

**Keywords:** Problem gambling, Sports betting, Betting, Football, Sport, Public health

## Abstract

Betting on the various codes of football in Australia accounts for the majority of sports betting, with Australian rules football (AFL) by far the most popular sport in Australia. Several studies have revealed the heavy presence of gambling advertising during AFL broadcasts, and a frequently used advertising strategy involves the use of well-known AFL commentators outlining their tips and betting suggestions. To date, no research has examined the hypotheses that skill may help in predicting AFL matches and monetary outcomes from AFL betting. Rather than merely discounting such ideas, it is important to test them empirically. The aims of this study were therefore, to examine if (1) expert AFL tipsters made better predictions than random picks, (2) expert AFL tipsters gained greater monetary reward than random selection, and (3) expert tipsters’ prediction accuracy improved with betting experience. To this end, six seasons of AFL matches, odds data, and expert tipster data were analysed retrospectively, totalling 1141 matches. Random selections were calculated for each match using an inbuilt random number generator within Microsoft Excel and a $2 simulated wager was applied for each AFL match. The results of mixed-effects modelling showed that experts picked more correct outcomes than random selection; experts’ correct predictions were partially mediated by home-game selections; no difference in monetary outcome was observed for experts compared to random selection; experts’ predictions did not improve over time. The results of this study may be used to inform both psychological interventions that target gamblers’ illusions of control, and public health gambling harm prevention messaging.

## Introduction

In Australia, the involvement with online gambling is growing, with rates having increased from less than 1% in 1998-99 to 8% in 2011 (Gainsbury et al., 2014, 2015), and total gambling expenditure (losses) increased by 5% from $23.694 billion in 2016–2017 to $24.887 billion in 2017–2018 (Queensland Government Statistician’s Office, 2019). Advances in technology and increased usage of electronic mobile devices over the past decade have changed the way in which gamblers engage with online gambling. The convenience of mobile electronic devices is generally understood to be a major contributor to the increase of online gambling (Gainsbury et al., 2015), and Australia has among the highest usage of smartphones in the world with approximately 88% of the population owning a smartphone (Deloitte, 2017).

The growth of online gambling has been implicated in the progression of sports betting (Lawn et al., 2020). Australia has seen a substantial increase in participation in sports betting in recent years (Armstrong et al., 2018) along with a significant increase in marketing for sports betting products (Deans et al., 2017). Betting on the various codes of football in Australia accounts for the majority of sports betting (Gainsbury & Russell, 2013), with Australian rules football (Australian Football League, or AFL) by far the most popular sport in Australia (Kyngäs et al., 2017). Several studies have noted the heavy presence of gambling advertising during AFL broadcasts (Gordon & Chapman, 2014; Milner et al., 2013; Thomas et al., 2012). A frequently used advertising strategy involves the use of well-known AFL commentators outlining their tips and betting suggestions (Milner et al., 2013). Milner et al. (2013) suggested that such tips tend to be presented with a sense of authority and confidence, which provides gamblers with further positive association with the likelihood of winning. Other researchers have likewise raised concern that sports betting advertising creates an inflated sense of control among bettors (Lopez-Gonzalez et al., 2018). Sports-bettors have been reported, in general, to struggle to understand the influence that chance has had in their betting history, and feel confused about the role their skills and knowledge have played (Lopez-Gonzalez et al., 2018). In a qualitative study of sports-bettors undergoing treatment for problem gambling (Lopez-Gonzalez et al., 2018), bettors described an awareness that their beliefs in their skills in being able to make accurate predictions were likely viewed as cognitive distortions by scientists and therapists; however, they still maintained a number of beliefs about their betting skills. The authors suggested that the existence of expert tipsters can reinforce such ideas (Lopez-Gonzalez et al., 2018).

Irrational beliefs about the ability to control the outcome of the game have long been considered an important factor in why some gamblers continue to play despite repeated losses (Gaboury & Ladouceur, 1989; Toneatto et al., 1997). Ladouceur et al. (1998) compared the results of expert horse gamblers with those derived from a random selection of bets. While experts made more correct selections, no differences were observed when comparing monetary outcomes, and in the long run, the outcomes of the bets were always negative (Ladouceur et al., 1998). There has been only limited research on whether knowledge and skill may play a part in the prediction of team or player sporting events. For example, teams playing at home have been reported to win significantly more games in the American National Football League, an advantage recognised by bettors (Vergin & Sosik, 1999). Further, player or team ranking have been shown to be useful predictors of basketball and tennis matches (Boulier & Stekler, 1999). In another study, expert hockey bettors made more correct predictions than chance, but, as Ladouceur et al. (1998) found with horse bettors, monetary outcomes were no better than random selection (Cantinotti et al., 2004). As suggested by Cantinotto et al. (2004), the gamblers’ illusions of control may be reinforced by their increased accuracy compared to chance, due to their greater selection of home games. Khazaal et al. (2012) compared the predictions of 10 consecutive football (soccer) match outcomes of the European Championship among football (soccer) experts, football amateurs, and laypersons (non-initiated to football). Expertise, age, or gender did not impact the accuracy of predictions, suggesting that any belief that knowledge and expertise can improve the accuracy of predicting soccer matches is an illusion of control.

To date, no research has examined the hypotheses that skill may help in predicting AFL matches and monetary outcomes from AFL betting. Rather than merely discounting such ideas, it is important to test them empirically. Given the emphasis on expert tipsters during AFL broadcasts, the outcomes of such research may have important implications for public health policy and messaging. The aims of this study were therefore to examine if expert AFL tipsters made better predictions than random selections, and whether this translated to monetary outcomes. To that end, we examined first, whether expert AFL tipsters predicted a greater number of correct AFL game outcomes than random selection. Second, if expert AFL expert tipsters gained greater monetary reward than random selection, and third, if expert AFL tipsters’ prediction accuracy improved with betting experience.

## Methods

Six seasons of AFL home-and-away games and odds data comprising 1141 matches between 24 and 2016 and 22 August 2021 were analysed retrospectively. The historical AFL results and odds data were sourced from an Australian sports betting website (Australia Sports Betting, 2021). Historical expert tipster data were sourced from a popular Australian sports opinion website, independent of sports betting firms, where expert tipsters present their tips for each weekly round along with expert commentary about each upcoming match (The Roar, 2021). Tipsters comprised freelance sports journalists and editors for a sports opinion website, sports journalists for two major daily newspapers, and sports journalists with a commercial television station. A total of 3442 bidding incidents were recorded, including tipsters’ prediction of the outcome of the game, the game outcomes (home win vs. loss), and the historical odds of winning for both teams. A $2 simulated wager was made by the researchers for each AFL match against each tipster and was calculated using Microsoft excel. Prediction of the game outcome for each of the 1141 matches (home team win vs. loss) representing random selection was generated in Microsoft Excel (random number generator function: RAND), with the $2 simulated wager applied as described above. The outcome of selecting winning matches and the amount of money returned for a $2 wager were recorded for the expert tipsters and the random selections.

### Statistical Analysis

Given that tipsters predicted varying number of games, the average proportion of correct predictions was estimated by pooling the Freeman-Tukey Double Arcsine transformed rates of correct prediction from the 14 tipsters with inverse-variance weights obtained from a random-effects model. The confidence intervals are based on score procedures (Nyaga et al., 2014).

The odds of tipsters correctly predicting the outcome of a game were compared to that of random selections using a mixed-effect logistic model with a random intercept per tipster and random selection. The binary variable indicating tipster or random selection is the singular independent variable in the regression model regressed against the binary variable of prediction outcome (correct vs. incorrect prediction). A mixed-effect approach was chosen to account for the potential correlated nature between predictions made from the same tipster.

The comparison between the average amount of money won by tipsters against that from random selection was carried out using a mixed-effect linear regression model. As with the logistic regression, the linear model had a random intercept per tipster and random selection. An additional analysis including only the information on the monetary gain was carried out (i.e., including only correctly predicted games). For this additional analysis, the monetary gain variable was logarithmically transformed to obtain more meaningful interpretations and to address the right skewness of the data. Normality assumptions were subjectively checked and confirmed using quantile-quantile plot of standardised residuals.

Moreover, to explore whether tipsters’ winning odds vary with increasing betting experience, a variable acting as a proxy of tipsters’ experience was created by sequentially numbering the chronologically ordered games predicted by each tipster. For example, if a tipster predicted 2 of the 1141 games, the proxy variable for the earlier game would be 1 and the later 2. This proxy variable as well as its interaction term with the binary variable indicating tipsters or random selections were added into the aforementioned random-effect logistic model. The impact of the betting experience and its interaction effect, which signalling whether the betting experience had any differential impact on the outcomes between tipsters and random selection, were assessed using likelihood-ratio tests between the nested models. Lastly, the mediation effect of home game tipping was examined using the four-step approach proposed by Baron and Kenny (1986).

All statistical analysis were performed in Stata version 17.0 (StataCorp). We used an alpha level of 0.05 for all tests (2 sided).

## Results

### Accuracy of Predictions

Tipsters correctly predicted 65% (95% CI 63%-68%) of AFL games compared to 49% (95% CI 46%-51%) by random selection. Results of the logistic regression revealed that, for a given tipster, the odds of correctly predicting the game outcome were 98% higher compared to random selections (OR = 1.98, 95% CI 1.64–2.39, *p* < 0.001).

### Monetary Outcomes

Results of the mixed-effect linear regression suggested that, on average, there was no significant difference in monetary outcomes between expert tipsters and random selections (7 cents greater monetary outcome among tipsters, 95% CI -5 to 18 cents). When considering correctly predicted games only, and with logarithmic transformation of the monetary gain variable, tipsters on average gained 41% less compared to random selection (*p* = 0.004, 95% CI 15.7–58.9%). Table 1. presents a summary of betting outcomes by tipster and randomly generated outcomes.

**Table 1:** Summary of betting outcomes by tipster and randomly generated outcomes

Tipster	Total number games	Total money gain(+)/loss(-)	Total number correct predictions	Correct prediction rate (95% CI)	Weight^†^ (%)
1	529	-76.20	334	0.63 (0.59–0.67)	10.80
2	198	-37.04	122	0.62 (0.54–0.68)	6.90
3	331	-50.46	205	0.62 (0.56–0.67)	9.00
4	529	-85.42	335	0.63 (0.59–0.67)	10.80
5	180	-23.80	113	0.63 (0.55-0.70)	6.60
6	156	-4.56	115	0.74 (0.66–0.80)	6.80
7	156	+ 3.10	115	0.74 (0.66–0.80)	6.80
8	471	-52.06	315	0.67 (0.62–0.71)	10.60
9	9	-2.82	6	0.67 (0.30–0.93)	0.60
10	126	-50.68	70	0.56 (0.46–0.64)	5.10
11	64	-9.52	39	0.61 (0.48–0.73)	3.10
12	189	-13.8	128	0.68 (0.61–0.74)	7.00
13	315	-31.64	208	0.66 (0.61–0.71)	9.00
14	189	-13.18	133	0.70 (0.63–0.77)	7.20
				Pooled average: 0.65 (0.63–0.68)	100
RNG	1141	-228.16	554	0.49 (0.46–0.51)	n/a

Note: †Inverse−variance weight for calculating the pooled average correct prediction rate; RNG, random number generator

### Accuracy of Predictions by Experience

Greater betting experience showed no statistically significant impact on tipsters’ betting outcomes (chi2 = 5.39, df = 2, *p* = 0.07), and such null effect was consistently identified among tipsters’ experience and random selections (chi2 = 3.37, df = 1, *p* = 0.07). Lastly, home game tipping appeared to have a partial mediation effect on the higher winning odds observed among tipsters.

## Discussion

The aim of the present study was to examine if knowledge about AFL is associated with a greater accuracy rate when picking the results of the games, and greater monetary outcomes when considering betting odds, than random selection. With that goal, we retrospectively compared the selections made by expert tipsters with those produced by a random selection procedure. The results showed that expert tipsters had a greater accuracy rate than random selection when picking the results of the games. However, from a financial perspective, selections made by expert tipsters did not achieve better monetary outcomes from the rate of return on a $2 wager than chance, and the overall return from a $2 bet was negative for random picks and for all but one of the tipsters. In fact, when considering only the monetary gains, experts gained significantly less on average per wager. The finding that expert tipsters had greater odds of making accurate predictions, yet the outcome of their bets were not significantly better than randomly selected choices, may be explained by the tendency for experts in this study, to select favourites as opposed to long odds. A similar finding was reported by Ladouceur et al. (1998), who found expert horse gamblers picked the winning horse more often than a random selection, with short odds more often selected by experts than long odds, but in the long run the outcome of the bets were not significantly better than those of regular gamblers or random selection.

These results suggest that monetary outcomes from gambling on AFL are not influenced by skill or knowledge about AFL. The data suggests that, while knowledge about AFL may assist gamblers in making accurate predictions of the outcome of games, for example, home game advantage, as indicated by the mediation effect of selecting home games, this does not improve the financial outcome beyond chance predictions when betting odds are involved. In other words, in the long run, making accurate predictions of the outcome of AFL games, and winning money by betting on AFL games, are not the same thing. This is an important distinction that gamblers may not fully appreciate. Previous research has suggested that sports bettors struggle to understand the role that chance and skill have in their gambling activity (Lopez-Gonzalez et al., 2021).

The finding that expert tipsters had higher odds of making correct predictions than randomly selected picks, despite no difference in financial outcomes, may explain why some gamblers continue to gamble despite losing money in the long term. Being right is a powerful reinforcer for humans; making accurate predictions is reinforcing. Functional magnetic resonance imaging (fMRI) studies indicate that making correct responses activates reward networks. For example, correctly recognising items in a memory retrieval task yields greater activation in the right anterior prefrontal cortex than incorrect responses (Buckner et al., 1998; Han et al., 2010), and this effect appears to be particularly influential among adolescents (Satterthwaite et al., 2012). Satterthwaite et al. (2012) used fMRI to demonstrate increased activity in the ventral striatum among adolescents, in response to correct compared to incorrect responses during a challenging cognitive task. These brain regions are similarly activated when engaging in pleasurable activities such as eating food or listening to music (Mas-Herrero et al., 2021). What happens when a person simultaneously makes a correct response but loses money? A recent study by Ruissen et al. (2018) using fMRI, examined the rewarding effect of being right, even in the case of a negative outcome (monetary loss). Increased striatal activations were found for gains compared to losses and for correct compared to incorrect responses. Further, enhanced striatal activity was observed for monetary losses that were preceded by correct compared to incorrect responses, and identical monetary outcomes showed enhanced activation in the striatum for outcomes following correct advices compared to incorrect advices (Ruissen et al., 2018). While a gambler does not lose money when they correctly predict an individual bet, overall, winnings typically are not enough to offset their losses in the longer term. The current study demonstrated expert tipsters experienced greater monetary losses yet had higher odds of making a correct prediction than random selection. The reinforcing effect of being right, in terms of intrinsic pride in ones’ cognitive abilities and the social context of watching sport and gaining ‘bragging rights’ with one’s friends, may explain why some gamblers continue to wager despite poor financial outcomes in the long term. In a cognitive model of problem gambling, the gambler continues to gamble because they overestimate their chances of winning money due to cognitive distortions (Ladouceur & Walker, 1996). The reinforcing effect of making correct selections may uphold AFL bettors’ distorted cognitive beliefs concerning their ability to win money in the long term.

Future research could examine AFL gamblers’ comprehension of the nature and construction of betting odds. Subsequently, gamblers, particularly younger groups, should be educated about the difference between making accurate predictions about an AFL game and winning money gambling on AFL games; that is, making accurate predictions and winning money gambling, in the long term, are not the same thing. The limited research on gambling harm prevention messaging suggests that messaging needs to be targeted rather than generalised messaging and should include informative content about gambling to increase health literacy (Riley et al., 2022). This is particularly important given the heavy presence of gambling advertising during AFL broadcasts (Gordon & Chapman, 2014) which commonly include betting tips by commentators (Milner et al., 2013). In that respect, questions should be raised on the appropriateness of AFL broadcasters to include expert tips intertwined with betting advertising.

In addition to informing public health messaging, the results of this study have clinical implications. Cognitive distortions have long been established as playing an important role in the maintenance of excessive and harmful gambling (Benhsain et al., 2004; Gaboury & Ladouceur, 1989; Toneatto et al., 1997). However, clinicians may find it difficult to help the gambler to challenge beliefs concerning the usefulness of knowledge and skill when betting on AFL, among clients who consider themselves experts in AFL. The results of this study could serve as a basis for addressing such illusions of control while clinicians deliver cognitive therapy, a point similarly made by Cantinotti et al. (2004) in their study of hockey gamblers. This education may include, for example, educating clients that they win less money than they lose because of the odds calculated by sports betting agencies.

A potential limitation of this study is that it was not performed under actual gambling conditions. For example, real gamblers may not necessarily place a bet on every game, nor wager the same amount of money on each game over the season. Real gamblers may wager more when they are confident of outcomes considering the odds offered. We do not know if this offsets the unfavourable financial outcomes achieved when the same amount is wagered consistently. Future research may replicate this study in a real gambling context, such as observing the outcomes of real-life wagering on AFL and comparing with randomly generated selections and/or lay persons not initiated to AFL. A further limitation is that multiple comparisons conducted in our analyses are not adjusted given the exploratory nature of this study. The results, therefore, should be considered with caution.

In conclusion, expertise in AFL was related to increased odds of correctly predicting the outcome of matches but had no influence on the financial outcome of a $2 wager compared to random picks. Therefore, the belief that skill and knowledge of AFL can improve betting outcomes should be considered a cognitive distortion. The present results may be used to inform both psychological interventions that target gamblers’ illusions of control, and public health gambling harm prevention messaging.

## Data Availability

Data sets generated during the current study are available from the corresponding author on reasonable request.
